# ENHANCING VISUOMOTOR INTEGRATION IN OLDER ADULTS: INSIGHTS FROM A MULTI-FACETED TRAINING PROTOCOL

**DOI:** 10.1093/geroni/igae098.3965

**Published:** 2024-12-31

**Authors:** Edward Ofori, Justin Foster

**Affiliations:** Arizona State, Phoenix, Arizona, United States; Excelling Edge, Phoenix, Arizona, United States

## Abstract

This investigation rigorously assessed the impact of an 8-week training regimen on visual-motor integration and balance in older adults, particularly focusing on individuals diagnosed with Parkinson’s Disease (PD). Seven participants over the age of 50 years (mean age: 62.4 ± 8.6 years for Control, 73.0 ± 5.7 years for PD) underwent pre- and post-intervention evaluations using the Senaptec Sensory Station and Sway Medical systems. The assessments targeted Eye-Hand Coordination (EHC), Multiple Object Tracking (MOT), and balance metrics. Participants engaged in weekly in-person training sessions and bi-weekly at-home exercises designed to enhance dynamic balance, visual-motor integration, and cognitive function. Each at-home session included activation (saccades, brisk walk, dynamic balance), dynamic vision (customized Senaptec app exercises), and a final phase tailored to individual needs, focusing on either eye-hand coordination or parasympathetic nervous system (PNS) activation. Results indicated no statistically significant improvements in balance assessments (p>0.10); however, significant improvements were observed in Central EHC within the PD group (t = 14.35, p = 0.04), with combined analysis across groups also showing significance (t = 2.73, p = 0.03). For peripheral EHC, the Control group exhibited significant task time improvements (t = 3.61, p = 0.02), with combined group analysis confirming significance (t = 3.14, p = 0.02). MOT scores showed the Control group outperforming the PD group (p < 0.02, 1712±605°/s vs 893±220°/s). The findings suggest that targeted visual-motor training can mitigate cognitive and motor declines in aging and neurodegenerative conditions, warranting further large-scale studies.

